# Isolation of bacteriophages specific to bovine mastitis-causing bacteria and characterization of their lytic activity in pasteurized milk

**DOI:** 10.14202/vetworld.2024.207-215

**Published:** 2024-01-25

**Authors:** Napakhwan Imklin, Patinya Patikae, Peekarn Poomirut, Pipat Arunvipas, Rujikan Nasanit, Somchai Sajapitak

**Affiliations:** 1 Department of Biotechnology, Faculty of Engineering and Industrial Technology, Silpakorn University, Nakhon Pathom, Thailand; 2 Veterinary Clinical Study Program, Faculty of Veterinary Medicine, Kasetsart University, Nakhon Pathom, Thailand; 3 Faculty of Veterinary Sciences, Mahasarakham University, Maha Sarakham, Thailand; 4 Department of Large Animal and Wildlife Clinical Sciences, Faculty of Veterinary Medicine, Kasetsart University, Nakhon Pathom, Thailand

**Keywords:** bovine, *Escherichia coli*, *Klebsiella pneumoniae*, mastitis, phage, *Staphylococcus*

## Abstract

**Background and Aim:**

Bovine mastitis is one of the most serious issues in dairy production. It is caused by contagious and coliform pathogens such as *Staphylococcus* spp., *Escherichia coli*, and *Klebsiella pneumoniae*. In addition, the emergence of drug-resistant bacteria raises urgent concerns in the field of drug treatment, thus requiring the exploration of alternative treatments. Bacteriophage therapy has been shown to be a promising alternative approach for the control of antibiotic-resistant pathogens. In this study, we aimed to isolate phages specific to contagious mastitis and coliform mastitis, characterize the isolated phages, and examine their ability to lyse bacteria in pasteurized milk samples.

**Materials and Methods:**

The *Staphylococcus* phage vB_Sau-RP15 isolated from raw milk in our previous study was used in this study. Other three phages, vB_Eco-RN12i1, vB_Kpn-RN14i1, and vB_Ssc-RN20i3, were isolated from wastewater using *E. coli* 5823, *K. pneumoniae* 194, and *Staphylococcus sciuri* MM01 as hosts, respectively. The host range and efficiency of plating (EOP) were determined following phage isolation. Moreover, the lysis activities of these phages against their hosts were investigated in pasteurized milk using a multiplicity of infections (MOIs) of 10 and 100 at 37°C. Phages were applied using individual and combination phages.

**Results:**

According to the EOP results, all phages showed high specificity to their respective hosts. They are tailed phages with distinct morphologies. Individual phage treatments in spiked pasteurized milk with their respective bacterial hosts significantly reduced the bacterial counts in both MOI conditions during the first 2 h of the treatment (approximately 1–8 log reduction compared to the control). Although these phages specifically infected only their hosts, the phage cocktail resulted in a better result compared to the use of individual phage. However, bacterial regrowth was observed in all experiments, which may be related to the development of phage-insensitive mutants.

**Conclusion:**

Our findings suggest that the application of phages could be used to treat bovine mastitis. Phage cocktail is suitable to promote the efficacy of phage treatment in pasteurized milk. However, when considering the use of phages in dairy cows, certain phage properties in raw milk and *in vivo* and *ex vivo* should be highlighted to ensure their effectiveness as biocontrol agents for bovine mastitis treatment.

## Introduction

Bovine mastitis, which is a common problem in the dairy sector, is a significant challenge due to its widespread incidence and subsequent global economic losses. This disease is primarily caused by pathogenic bacteria that enter the mammary gland through the lock hole on the teat [1]. On the basis of the ecological niche from which the infection occurs, mastitis-causing pathogens can be divided into cow-adapted pathogens or environmental-bound pathogens. Cow-adapted mastitis pathogens primarily propagate in or on the cow’s udder. Examples include *Streptococcus agalactiae* and *Staphylococcus aureus*, which are usually transmitted from infected udders to uninfected udders by direct contact with the milker and milking machine during milking. Environmentally bound mastitis pathogens, such as coliform bacteria such as *Escherichia coli* and *Klebsiella* spp., *Enterococcus* spp., and *Streptococcus* spp. (e.g., *Streptococcus uberis*), mainly propagate in the dairy stable [2, 3]. The majority of infected cows are symptomatic and the physical properties of milk have changed. Environmental mastitis often occurs in farms with inadequate hygiene practices [4]. Clinical signs were used to categorize the infected cows. A cow with clinical mastitis has physical abnormalities in the udder and milk, as well as certain systemic symptoms. The other is subclinical mastitis with normal milk and udder. The somatic cell count increased in two of these cases [5]. Due to the low quality of bulk milk mixed with some mastitis milk, farmers face financial challenges due to decreasing prices and discarding milk containing antibiotics in treated cows [6].

The use of appropriate milking hygiene can prevent and control mastitis. Infected cows are generally treated with antibiotics, either intramammary and/or systemically. Various antibiotics are distributed worldwide; however, improper use of antibiotics, such as inapplicable doses and durations, leads to unsuccessful mastitis control and the development of antimicrobial-resistant bacteria. Intramammary antibiotics are increasingly being used and guidelines have been established to ensure their appropriate use in preventing and controlling mastitis [7, 8]. The use of antibiotics as a treatment for bovine mastitis has the disadvantages of potential antibiotic residues in milk, which raises concerns regarding the safety of food products [9]. In addition, the emergence of multidrug-resistant bacteria has become an obstacle in the fight against mastitis-causing infections [10]. There are several alternative treatments for bovine mastitis, such as bacteriophages (phages), phage-derived enzymes, probiotics, vaccination, nanoparticles, cytokines, natural compounds, and herbs. Bacteria, including mastitis pathogens, are known to form biofilms to protect themselves from adverse conditions and chemical damage, which is one of the biggest challenges of antibiotic treatment. Phages and their hydrolytic enzymes have been reported to be an advantageous strategy to eliminate biofilms. In addition to antibacterial activity, other approaches provide immunomodulatory and anti-inflammatory effects at an economical cost compared to antibiotics [11–16]. The main objective of this research was the use of phages against mastitis pathogens.

Phages infect and lyse only bacterial cells, and they are viruses. High specificity for hosts of phages is considered to be a favorable characteristic, as they do not attack beneficial bacteria. However, this narrow host range necessitates some strategies to enhance phage efficiency. Phage cocktails have recently become an attractive solution. The use of phage cocktails would be advantageous for clinical treatment if the initial infection was detected and pathogens could not be identified. Phage therapy for bovine mastitis has recently been proposed and intensively studied. A number of *in vitro* studies have reported the use of phages to control mastitis pathogen. For example, a staphylococcal phage has been used to eliminate methicillin-resistant strains of *S. aureus* [17]. Another study examined the phage lytic potential against drug-resistant *S. aureus* isolated from milk collected from teats with mastitis. Isolated phages have demonstrated the ability to mitigate *S. aureus* growth [18]. Hamza *et al*. [19] reported the capability of the phage SA to suppress the growth of multidrug-resistant *S. aureus* isolated from mastitis milk for 8 h. In another study, *S. aureus* was isolated from subclinical bovine mastitis milk and assessed for its susceptibility to antibiotics and phages. These *S. aureus* isolates resisted several drugs but were significantly susceptible to phages [20]. On the other hand, the literature on phages against contagious mastitis pathogens *E. coli* and *S. agalactiae* is scarce [21–23]. In mouse mastitis models, phages have been reported to intramammarily reduce the number of propagating pathogens [24–27]. A study that treated asymptomatic mastitis in cows infected with *S. aureus* using *Staphylococcus* phage K reported a 16.7% cure rate (3 of 18 quarters) [28], whereas another study reported a cure rate of 66.66% (4 of 6 cases) when a phage cocktail consisting of *Bacillus*- and *Escherichia*-specific phages was applied to symptomatic mastitis cows [29]. The use of phages to treat mastitis is not straightforward due to proteases produced by the innate immune system and lipid and protein contents of raw milk that prevent phages from attaching to host cells [30].

Therefore, this study aimed to isolate specific mastitis-associated phages and characterize their lysing ability in pasteurized milk individually and in a cocktail to complement the existing arsenal of phages for bovine mastitis.

## Materials and Methods

### Ethical approval

The study was approved by the Institutional Animal Care and Use Committee, Kasetsart University, Bangkok, Thailand (ACKU64-VET-037).

### Study period and location

This study was conducted from June 2020 to December 2021 at the Faculty of Veterinary Medicine, Kasetsart University, Thailand, and the Department of Biotechnology, Faculty of Engineering and Industrial Technology, Silpakorn University, Nakhon Pathom, Thailand.

### Bacterial culture

Twelve bacterial isolates were used in this study (Table-1). *S. aureus* NP01 isolated from raw milk was obtained from Kasetsart University Veterinary Teaching Hospital, Nong Pho, Thailand. Other 11 bacterial isolates, which were all bovine mastitis field isolates, were provided by Kamphaeng Saen Veterinary Diagnostic Center, Kasetsart University. All bacteria were grown overnight in tryptic soy broth (TSB) (HiMedia, India) at 37°C, transferred to a fresh medium, and incubated at 37°C to obtain the mid-exponential phase culture before performing experiments.

**Table-1:** EOP of isolated phages tested on mastitis-associated pathogens.

Bacterial strain	EOP value			

	vB_Sau-RP15	vB_Ssc-RN20i3	vB_Eco-RN12i1	vB_Kpn-RN14i1
Beta-hemolytic *E. coli* 1154-1	−	−	−	−
Beta-hemolytic *E. coli* 1154-2	−	−	−	−
*E. coli* 1033	−	−	−	−
*E. coli* 1077	−	−	−	−
*E. coli* 1100	−	−	−	−
*E. coli* 5823	−	−	1 (host)	−
*E. coli* 5838	−	−	−	−
*K. pneumoniae* 194	−	−	−	1 (host)
*S. aureus* NP01	1 (host)	−	−	−
*S. sciuri* MM01	−	1 (host)	−	−
*Staphylococcus* spp. 1172	0.004	−	−	−
*Staphylococcus* spp. 1182	−	−	−	−

EOP=Efficiency of plating, host=Isolated host, EOP > 0.5 (high efficiency), EOP < 0.001 (inefficiency), −=Not specific. *E. coli=Escherichia coli, K. pneumoniae=Klebsiella pneumoniae, S. aureus=Staphylococcus aureus,* *S. sciuri=Staphylococcus sciuri*

### Bacteriophage isolation and preparation

Wastewater samples were collected from bovine farms in Nakhon Pathom, Thailand, and areas outside the farms within a distance of <10 m. To isolate the phages, the wastewater samples were centrifuged at 5000× *g* for 10 min at 25°C to remove large particles. The supernatant of each sample (45 mL) was then mixed with 10× TSB and a bacterial host strain in a ratio of 9:1:0.1. The mixture was statically incubated at 37°C overnight and then centrifuged at 10,000× *g* for 10 min at 25°C. For initial phage screening, 100 μL of overnight bacterial culture was added to 3.5 mL of molten tryptic soy agar (TSA) (0.45% agar) and then poured onto a TSA plate. Ten microliters of the supernatant was applied to the solidified bacterial lawn. After overnight incubation at 37°C, only those samples with lysis clearance were selected for phage isolation and purification. Plaques were produced using the agar overlay technique for phage purification. Briefly, 100 μL of diluted crude phage sample was mixed with 100 μL of each overnight bacterial culture and 3.5 mL molten TSA. The mixture was poured onto a TSA plate and incubated overnight at 37°C. Different plaque morphologies were observed using sterile cutting tips. The selected plaques were immersed in 1000 μL of SM buffer for 1 h. The agar overlay method was then performed for further phage purification. At least, five rounds of purification were performed. High-titer phage stocks were then prepared. In brief, each phage suspension was subjected to the agar overlay method to obtain confluence lysis. We collected the top agar with numerous plaques in 15-mL centrifuge tubes. The remaining agar residue was washed out with TSB (2–3 mL) and then poured into the same tubes. The tubes were centrifuged at 10,000× *g* for 15 min at 25°C, and the supernatant was filtered through 0.22 μm polyethersulfone (PES) filters (Johnson Test Papers, UK) before being kept at 4°C as phage stock at a titer of 10^9^–10^10^ plaque-forming units per milliliter (PFU/mL).

### Host range and efficiency of plating (EOP) determination

Twelve bacterial isolates (Table-1) were used to explore the phage host range. The spot technique was carried out by applying 10 μL of each phage (10^8^ PFU/mL) to each bacterial lawn. Plates were incubated overnight at 37°C. Spot zones were observed and recorded as + for clear zones and − for no lysis zone. To assess the relative efficacy of phage infectivity against other bacterial strains and the bacterial host, the EOP analysis was conducted using the agar overlay method. The EOP values were calculated by dividing the phage titer obtained from the tested bacterial isolates by that obtained from the bacterial host. EOP values reveal phage efficiency according to the following scores: EOP > 0.5 as high effective, 0.5 > EOP < 0.2 as medium effective, 0.2 > EOP < 0.001 as low effective, and EOP 0.001 as ineffective. Bacterial isolates that were insensitive to the tested phages were defined as not specific (−).

### Morphology

High-titer phage suspensions were deposited on glow-discharged formvar-coated copper grids and allowed to adsorb for 10 min. Grids were negatively stained with 2% uranyl acetate. Phage images were captured using an HT7700 transmission electron microscope (Hitachi, Japan) operated at an electron acceleration voltage of 80 keV. Phage particles (n = 10) from each phage isolate were measured using ImageJ 1.54d software (http://imagej.org).

### Application of individual phages to pasteurized milk

This experiment was divided into three groups: Phage treatments at a multiplicity of infection (MOI) of 1 and 100 and a bacterial control group. For phage treatments, 3 mL of the phage suspension was added to 27 mL of pasteurized full-fat milk containing bacteria (10^7^ colony forming units [CFU]/mL) to obtain a final titer of 10^7^ PFU/mL and 10^9^ PFU/mL for MOI 1 and 100, respectively. For bacterial control, a mid-log phase host was added to pasteurized milk to obtain a final titer of 10^7^ CFU/mL without adding phage. Pasteurized milk was then incubated at 37°C under shaking at 120 rpm for 6 h. Aliquots were collected every hour to determine the bacterial and phage counts. For bacterial counts, the samples were serially diluted with 0.9% sodium chloride and spotted onto TSA plates. Plates were incubated at 37°C for 16–18 h. Colonies were counted after incubation and calculated as CFU/mL. For phage enumeration, the samples were immediately centrifuged at 10,000× *g* for 4 min and filtered through 0.22 μm PES syringe filters. The plaques were enumerated using the agar overlay method, and the phage titer was calculated as PFU/mL.

### Phage cocktail application in pasteurized milk

Four phages (vB_Sau-RP15, vB_Eco_RN12i1, vB_Kpn_RN14i1, and vB_Ssc_RN20i3) were combined to combat *S. aureus* NP01 and *E. coli* 5823 with an MOI of 100. We divided the experiment into three groups: Bacterial control, individual phage treatment, and phage cocktail treatment. Each mixture consisted of 27 mL of pasteurized full-fat milk containing bacteria (10^7^ CFU/mL) and 3 mL of phage cocktail (10^9^ PFU/mL for each phage). The experiment was conducted as stated in the previous section “Application of individual phages to pasteurized milk”. Samples for bacterial and phage enumeration were collected as previously described at 1-h intervals for 6 h.

### Statistical analysis

The application experiments were performed in triplicate, and the results were statistically analyzed by analysis of the variance (ANOVA) in bacterial titer across different groups and time points, as well as the phage titer. Statistical analyses were performed using the R V 4.1.1 (https://cran.r-project.org/) and Rstudio V 1.4.1717 (https://posit.co/download/rstudio-desktop/) programs with the repeated measure linear mixed model. The model was built using the “lmer” command line derived from package lmer4. We tested the model using the “ANOVA” command line. The “emmean” command line was used in the *post hoc* multiple comparison test [31].

## Results

Phage vB_Sau-RP15 has been isolated from raw milk using *S. aureus* NP01 as a host and characterized in our previous study [32]. Other phages, including vB_Eco-RN12i1, vB_Kpn-RN14i1, and vB_Ssc-RN20i3, were isolated in this study using *E. coli* 5823, *K. pneumoniae* 194, and *Staphylococcus sciuri* MM01 as hosts, respectively. The host range and EOP were determined following phage isolation. Most phages were specific only to their respective hosts (Table-1). Only the vB_Sau-RP15 phage was able to infect *Staphylococcus* spp. 172, another tested bacterial isolate. However, the efficiency was low (EOP = 0.004).

A reduction in bacterial titer and an increase in phage titer were observed when the bacterial hosts were challenged separately with the individual phages in pasteurized milk, as shown in Figure-2. vB_Sau-RP15 significantly diminished *S. aureus* NP01 at MOIs 1 and 100 compared with the control group (p < 0.01) (Figure-2a). Host titers increased in the control group but decreased when phages were present at both MOIs. It was shown that the phage reduced its host significantly at MOI 100 compared with MOI 1 (p < 0.01). The vB_Sau-RP15 titer at MOI 1 gradually increased to approximately 2 log PFU/mL, whereas at MOI 100, the titer increased insignificantly (0.3 log PFU/mL) (p < 0.01) at the end of the experiment (Figure-2b). When *E. coli* 5823 was artificially contaminated with pasteurized milk, its specific phage, vB_Eco-RN12i1, effectively killed the bacteria within the first 2 h (p < 0.01) (Figure-2c). The bacteria continuously regrew; however, at the end of the experiment, the *E. coli* loads in the treatment groups were approximately 3.4 log CFU/mL below the control group. An increase in phage titer was clearly observed at MOI 1, whereas at MOI 100, the phage titer increased slightly only in the 1^st^ h (Figure-2d). For *K. pneumoniae* 194, a significant reduction in bacterial titer was detected at the 1^st^ h, but there was no difference between bacterial titers at both MOIs during the test, except at the 6^th^ h (Figure-2e). Bacterial regrowth was continuously observed from the second to the final hour. The vB_Kpn-RN14i1 titer increased by approximately 3.6 and 1.1 log PFU/mL within the 1^st^ h at MOIs of 1 and 100, respectively (Figure-2f). However, the phage titer fluctuated slightly until 6 h. Notably, a significant increase in phage amplification was observed at MOI 1 in all experiments. In the case of vB_Ssc-RN20i3, it was observed that this phage exhibited the ability to decrease the number of *S. sciuri* MM01 within the initial hour of the experiment (Figure-2g). During the 2^nd^ h, the bacterial titer was reduced by 2.2 log CFU/mL, whereas that of the control group was 7.8 log CFU/mL. *S. sciuri* MM01 regrowth at MOI 100 was significantly observed at the 3^rd^ h compared with that at MOI 1 (p < 0.01) and continued for 6 h at both MOIs. The phage titer at MOI 1 increased sharply from the first to the 2^nd^ h and remained stable until the end (Figure-2h). On the other hand, at MOI 100, the amount of vB_Ssc-RN20i3 fluctuated from the second to the 6^th^ h.

**Figure-1:**
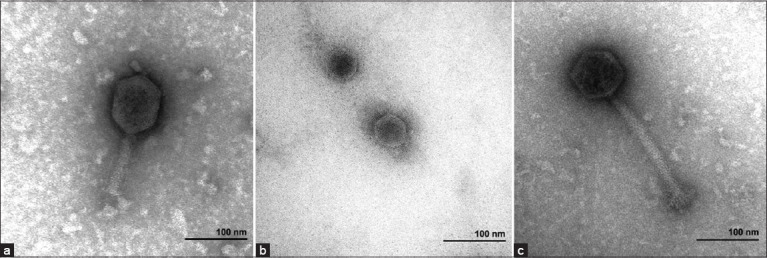
Transmission electron micrographs of phages (a) vB_Eco-RN12i1, (b) vB_Kpn-RN14i1, and (c) vB_Ssc-RN20i3.

The three isolated phages in this study had different appearances according to the TEM micrographs (Figure-1). vB_Eco-RN12i1 had a prolonged head and a contractile tail with a sheath around the tail tube. The head diameter was 87.24 ± 0.72 nm wide and 95.86 ± 1.56 nm long. The long contractile tail was approximately 105.43 ± 2.48 nm. vB_Kpn-RN14i1 had an icosahedral head and a short tail. The head had diameters of approximately 50.81 ± 0.81 and 52.98 ± 1.24 nm, while the tail was approximately 7.71 ± 0.18 nm long. vB_Ssc-RN20i3 had a symmetrical icosahedral head with 80.07 ± 2.72 nm in width and 78.56 ± 2.00 nm in length. This phage had a long, non-contractile tail with 183.96 ± 4.16 nm in length.

**Figure-2:**
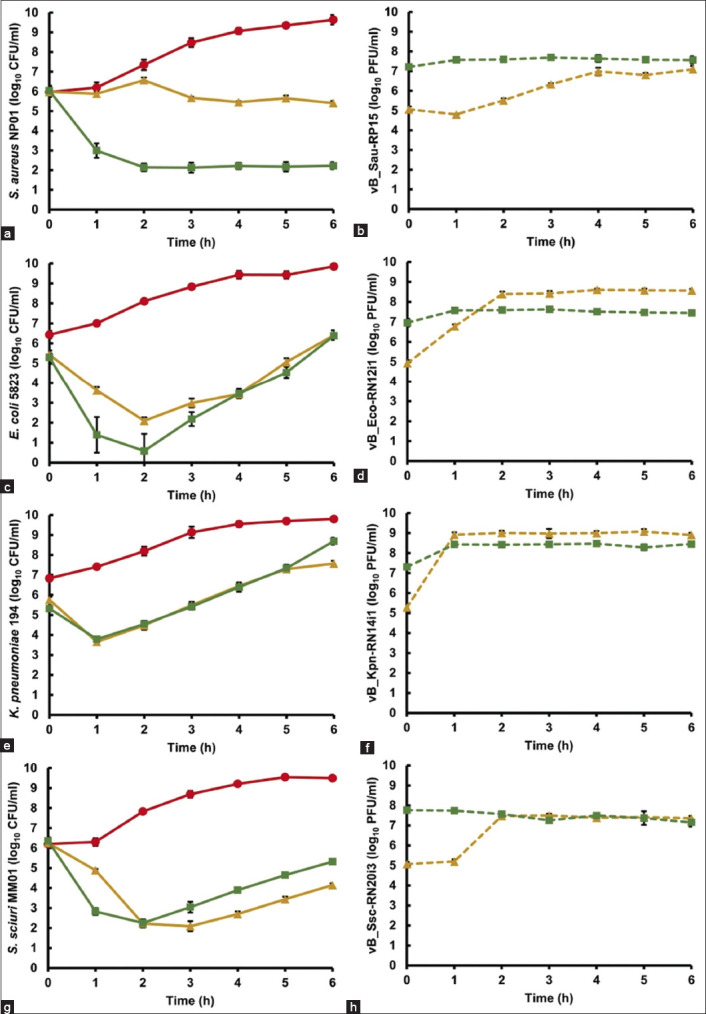
Single phage application in pasteurized milk against their specific hosts. (a) *S. aureus* NP01 count, (b) vB_Sau-RP15 count, (c) *E. coli* 5823 count, (d) vB_Eco-RN12i1 count, (e) *K. pneumoniae* 194 count, (f) vB_Kpn-RN14i1 count, (g) *S. sciuri* MM01 count and (h) vB_Ssc-RN20i3 count. The symbols represent different conditions following bacterial control (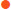 red), MOI 1 (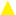
yellow), and MOI 100 (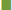 green). The error bars are derived from the standard deviation (SD) of three repeated experiments. *S. aureus*=*Staphylococcus aureus*, *E. coli*=*Escherichia coli*, *K. pneumoniae*=*Klebsiella pneumoniae*, *S. sciuri*=*Staphylococcus sciuri*, MOI=Multiplicity of infection.

**Figure-3:**
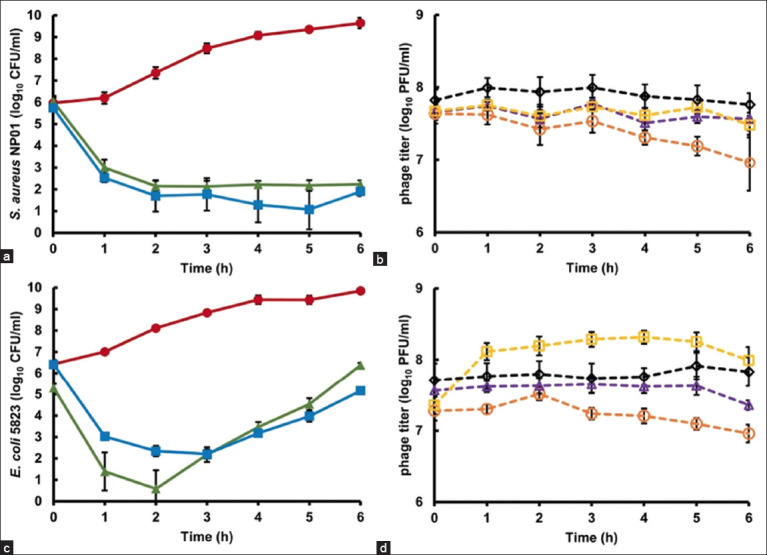
Cocktail phage application in pasteurized milk against *S. aureus* NP01 and *E. coli* 5823 at MOI 100. (a) *S. aureus* NP01 count, (b) phage counts for cocktail phage against *S. aureus* NP01, (c) *E. coli* 5823 count and (d) phage counts for cocktail phage against *E. coli* 5823. The symbols represent different conditions for bacterial counts, as follows: bacterial control (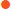 red), individual phage treatment (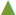 green), and phage cocktail treatment (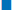 blue). The symbols represent different phage counts, as follows: vB_Sau-RP15 titer (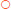 orange), vB_Eco-RN12i1 titer (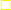 yellow), vB_Kpn-RN14i1 titer (◊ black), and vB_Ssc-RN20i3 titer (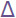 purple). The error bars are derived from the standard deviation (SD) of three repeated experiments. *S. aureus*=*Staphylococcus aureus*, *E. coli*=*Escherichia coli*, MOI=Multiplicity of infection.

The cocktail phages were similarly tested against *S. aureus* and *E. coli*. The reduction of the bacterial titer and the increase of the phage titer was observed similarly to the individual phage treatments. *S. aureus* was significantly reduced by both single and cocktail phages (p < 0.01) (Figure-3a). Bacterial titers decreased until the 5^th^ h, and regrowth was observed in the last hour following the phage cocktail treatment. On the other hand, no bacterial regrowth was observed in the single-phage condition. The number of each phage did not obviously increase (Figure-3b). For *E. coli* 5823, only vB_Eco-RN12i1 significantly reduced *E. coli* 5823 compared with cocktail phages at the first 2 h (p < 0.01) (Figure-3c). A slight increase in vB_Eco-RN12i1 was observed during the 1^st^ h, whereas other phages did not propagate (Figure-3d). However, the bacteria began to grow from the 3^rd^ h until the end of the test, without an increase in phage titer.

## Discussion

This study investigated the efficacy of bacteriophages (phages) against *S. aureus* NP01, *E. coli* 5823, *K. pneumoniae* 194, and *S. sciuri* MM01 which were isolated from bovine mastitis. The aim of this study was to assess their potential for mastitis control in spiked pasteurized milk. This would be beneficial for future phage utilization in the relevant field. The phage specifically targeting *S. aureus* demonstrated its ability to decrease the load of *S. aureus* NP01 in pasteurized milk when compared to the control group. In addition, phage concentration has an impact on the efficiency of phage activity. The ability of another phage to reduce *S. aureus* in heat-treated milk within 6 h was previously reported [33]. Another report revealed that the use of mixed phages, STA1.ST29, EB1.ST11, and EB1.ST27, could similarly eliminate *S. aureus* 7142 and *S. aureus* 10614 in pasteurized milk within 2 h for 98.8% and 98.2%, respectively [34]. However, in both studies, the efficiency of these phages was unsatisfactory when *S. aureus* was encountered in raw milk. It is possible that fat globules affect phage infection. Although, in our study, the phage was not applied to raw milk but to full-fat pasteurized milk, the lysis activity of vB_Sau-RP15 was positively effective as a bio-bactericide. vB_Sau-RP15 was isolated from raw milk; however, STA1.ST29, EB1.ST11, and EB1.ST27 phages were isolated from non-milk samples [34], which may result in differences in the lytic potential of these phages.

At the end of the experimental period, the vB_Sau-RP15 titer at MOI 1 was higher than that at MOI 100. A high phage-to-host ratio induces phage-insensitive mutants. During phage multiplication, the host cells underwent evolutionary changes that confer resistance against the phages, ultimately leading to phage-resistant bacteria [35]. In addition, several parameters have been shown to influence the phage propagation process. These factors include initial host titer, initial phage titer, phage adsorption ability, and phage propagation time [36]. In an ideal scenario, the phage particles would be able to infect all bacterial cells at a MOI of 100. This suggests that the presence of a relatively small number of bacterial cells within a significantly larger number of phage particles was insufficient for the successful proliferation of the phages. This is in contrast to the scenario where an equal number of bacterial cells and phages were present at MOI 1.

Not only contagious pathogens can cause mastitis, but coliform bacteria are also associated with this disease. *E. coli* and *K. pneumoniae* have been identified as causative agents of both clinical and subclinical mastitis in bovines, resulting in significant negative impacts on milk supply [37, 38]. Our study demonstrated that vB_Eco-RN12i1 had the ability to control *E. coli* 5823 in pasteurized milk. McLean *et al*. [39] demonstrated the efficacy of a phage cocktail in reducing the population of three different strains of *E. coli* in both ultrahigh-temperature-treated and raw milk within 3 h. Experiments were conducted at 25°C, and a MOI of 10,000 was used. Regrowth of *E. coli* ATCC 25922 and O127:H6 was not observed. On the other hand, the regrowth of *E. coli* O5:H- occurred at the 144^th^ h in raw milk. Porter *et al*. [23] successfully used a phage cocktail against *E. coli* originating from mastitis in raw milk. It should be noted that these phages have been isolated from a local sewage treatment plant. Grygorcewicz *et al*. [40] revealed the lytic capabilities of the ECPS-6 phage against *E. coli* O157:H7 A-2 in raw milk, observing no bacterial regrowth at MOI 50. However, in our study, *E. coli* 5823 regrowth has been observed since the 3^rd^ h, indicating the rapid evolution of the tested bacteria. This phenomenon was also observed when vB_Kpn-RN14i1 was applied to pasteurized milk artificially contaminated with *K. pneumoniae* 194. The use of *K. pneumoniae*-specific phages in milk is limited. Phage vB_KpP_HS106 was isolated from sewage using *K. pneumoniae* 106 as its host [41]. It had the capacity to reduce the host population in sterile skim milk within 6 h at MOIs 10 and 100. However, bacterial regrowth was observed at both MOI values.

*S. sciuri* has been reported as one of the coagulase-negative *Staphylococcus* associated with bovine mastitis. Some isolates have been found to carry drug-resistance genes [42]. Makky *et al*. [43] demonstrated the lytic activity of an interesting phage isolated from raw milk. It was able to eradicate multidrug-resistant *S. sciuri*, inhibit biofilm formation, and mitigate biofilm formation. Despite being classified as a temperate phage, this particular phage exhibits favorable characteristics that make it a potentially effective bacterial biocontrol agent. In the present study, although vB_Ssc-RN20i3 was able to minimize *S. sciuri* MM01 in pasteurized milk within the 1^st^ h, the bacterial concentration increased over the course of the trial. There was a decrease in phage titer at MOI 100 and a reduction in bacterial titer. This result implies that a limited number of bacterial cells were engaged in interactions with several phage particles, which resulted in rapid cell lysis without the subsequent generation of new phage progeny [44].

In this study, the phage cocktails consisted of four different phages specific to different mastitis pathogens. It should be noted that various bacterial strains may cause mastitis in cows. Therefore, the use of a mixed phage product is ideal for phage treatment. However, combined phages should not interfere with the effectiveness of phage treatment. Compared with the single-phage and phage cocktail treatments, the phage cocktail exhibited a significantly higher decrease in bacterial counts compared to the single-phage treatment. Although phages in this study were only specific to their respective hosts, utilization of a phage cocktail consisting of all phages against a bacterial strain yielded favorable outcomes compared to the single-phage condition. This may be because phages possess the potential to acclimate to diverse ecological niches, such as environments with various bacterial species and/or distinct phage populations, thereby initiating the evolution of microorganisms [45]. Therefore, phage cocktails are being developed to enhance host range expansion and lysis capabilities [46, 47].

It was evident from all the experimental findings that bacterial regrowth was observed at both MOIs. These results suggest that the ratio between phages and bacteria, as well as bacterial individuality, could potentially affect the treatment. At an MOI of 100, high phage numbers were introduced with the potential rise of resistant or insensitive bacteria that could replicate more efficiently, leading to fewer phages and high regrowth. These observations indicate that the bacterial host has undergone evolutionary changes to develop resistance to phage invasion [35].

## Conclusion

The efficiency of phages in controlling infections is influenced by various factors, such as bacterial and phage characteristics, phage-bacteria ratio, and environmental conditions under which they are challenged. The latter is particularly important in the case of an application for bovine mastitis. In the present study, we observed that all phages exhibited the ability to reduce the population of their respective bacterial hosts in full-fat pasteurized milk. Nevertheless, the phenomenon of bacterial regrowth was frequently observed, and it was attributed to phage resistance. To circumvent this problem, the utilization of phage cocktails has been proposed to improve the efficacy of phage therapy. The use of the isolated phages in this study following a cocktail strategy resulted in positive outcomes in terms of an increase in biocontrol activity. Hence, it is imperative to conduct a comprehensive investigation of raw milk as well as *in vivo* and *ex vivo* treatment modalities to evaluate the potential of isolated phages for bovine mastitis application. Nevertheless, this study further demonstrates that bacteriophages offer a feasible, future strategy to complement or replace existing antibiotics used to control and prevent bovine mastitis.

## Authors’ Contributions

SS, RN, PA, PaP, and NI: Conceived and designed the study. PaP, NI, and PeP: Performed the experimental works. PaP: Analysed and interpreted the data. NI and PeP: Drafted the manuscript. NI, PaP, and RN: Revised the manuscript. All authors have read, reviewed, and approved the final manuscript.
